# Real-time detection of Staphylococcus aureus transmission in hospitals

**DOI:** 10.1017/ash.2025.228

**Published:** 2025-09-24

**Authors:** Kristine Rabii, Courtney Takats, Gregory Putzel, Alice Tillman, Magdalena Podkowik, Julia Shenderovich, Natalia Argüelles, Anusha Srivastava, Alejandro Pironti, Sarah Hochman, Audrey Renson, Bo Shopsin

**Affiliations:** 1 NYU Langone Health; 2 NYU Langone; 3 NYU School of Medicine; 4 N/A; 5 NYU Langone Department of Microbiology; 6 NYU Langone Medical Center

## Abstract

Genomic surveillance of Staphylococcus aureus in hospitals usually focuses on clinical infections, missing transmissions from asymptomatic carriers and delaying detection and timely intervention. To address the issue, we performed whole-genome sequencing (WGS) on over 5,000 S. aureus isolates obtained from colonization screens at admission, in addition to standard clinical cultures, at two interconnected urban hospitals. By integrating genomic data with timestamped location information, we identified hundreds of transmissions missed by standard methods. However, nearly 70% of transmissions were detected during readmission after the index case had been discharged. This finding indicates that even with dense genomic sampling, real-time detection remains challenging due to asymptomatic carriage. Therefore, effective monitoring of nosocomial S. aureus transmission will likely require WGS and colonization sampling at both admission and discharge. The data also highlight patient- and strain-specific factors, including methicillin resistance, as predictors of S. aureus spread, which may enable cost-effective, targeted sequencing surveillance strategies.

